# The frequency-following response in late preterm neonates: a pilot study

**DOI:** 10.3389/fpsyg.2024.1341171

**Published:** 2024-05-09

**Authors:** Teresa Ribas-Prats, Sonia Arenillas-Alcón, Silvia Irene Ferrero Martínez, Maria Dolores Gómez-Roig, Carles Escera

**Affiliations:** ^1^Brainlab–Cognitive Neuroscience Research Group. Department of Clinical Psychology and Psychobiology, University of Barcelona, Barcelona, Spain; ^2^Institute of Neurosciences, University of Barcelona, Barcelona, Spain; ^3^Institut de Recerca Sant Joan de Déu, Esplugues de Llobregat, Barcelona, Spain; ^4^BCNatal–Barcelona Center for Maternal Fetal and Neonatal Medicine (Hospital Sant Joan de Déu and Hospital Clínic), University of Barcelona, Barcelona, Spain

**Keywords:** speech-ABR, FFR, infants, preterm, language

## Abstract

**Introduction:**

Infants born very early preterm are at high risk of language delays. However, less is known about the consequences of late prematurity. Hence, the aim of the present study is to characterize the neural encoding of speech sounds in late preterm neonates in comparison with those born at term.

**Methods:**

The speech-evoked frequency-following response (FFR) was recorded to a consonant-vowel stimulus /da/ in 36 neonates in three different groups: 12 preterm neonates [mean gestational age (GA) 36.05 weeks], 12 “early term neonates” (mean GA 38.3 weeks), and “late term neonates” (mean GA 41.01 weeks).

**Results:**

From the FFR recordings, a delayed neural response and a weaker stimulus F_0_ encoding in premature neonates compared to neonates born at term was observed. No differences in the response time onset nor in stimulus F_0_ encoding were observed between the two groups of neonates born at term. No differences between the three groups were observed in the neural encoding of the stimulus temporal fine structure.

**Discussion:**

These results highlight alterations in the neural encoding of speech sounds related to prematurity, which were present for the stimulus F_0_ but not for its temporal fine structure.

## Introduction

According to the World Health Organization (2023) preterm infants are those born at less than 37 weeks of gestation, with an incidence of around 1 in 10 babies of all births. Preterm birth is the leading cause of death in children under 5 years of age. However, the survival rates are increasing (Volpe, 2019) and with them the morbidities attributable to the immaturity of several organs, such as lungs, kidneys and brain (Swamy et al., 2008). Preterm infants are at increased risk for language, cognitive, sensory, and motor deficits (Hee Chung et al., 2020), and numerous studies have highlighted language and social interaction skills as the major areas affected compared to infants born at term (Barre et al., 2011; Nazzi et al., 2015; Carter and Msall, 2017).

Regarding language, impairments were observed in both expressive and receptive domains, as well as in word retrieval (Vohr, 2014; Palumbi et al., 2018). These language impairments have been associated with poor academic performance, and poor social, behavioral and emotional functioning across the life span (Cusson, 2003; Wolke et al., 2008; Howard et al., 2011; Dong et al., 2012; Stipdonk et al., 2018). Thus, a clear effort is necessary to identify neonatal biomarkers which make possible to understand the neurobiological basis of early language impairments associated with preterm births. Such biomarkers may promote early interventions, thereby reducing the negative neurodevelopmental consequences of prematurity.

Recent studies have used cranial ultrasonography (CUS) and magnetic resonance imaging (MRI) to characterize the anatomical underpinnings of language impairments in full-term and preterm neonates. Several MRI studies based on diffusion tensor imaging (DTI) observed that microstructural changes in cerebral white matter at the school-aged period were associated with language impairments in preterm and full term birth (Travis et al., 2016; Dodson et al., 2018). Greater increases in axonal diffusivity of the left posterior thalamic radiation from term-equivalent postmenstrual age to age 4 years was associated with poorer receptive and expressive language ability at age four (Young et al., 2017). Higher Mean diffusivity (MD) in the centrum semiovale and left superior temporal gyrus has also been linked to poorer language outcomes in preterm children (Aeby et al., 2013; Pogribna et al., 2014). Consistent with these early childhood findings, alterations in the uncinate fasciculus, splenium of the corpus callosum, and anterior commissure have been linked to language outcomes among preterm adolescents (Northam et al., 2012).

Since speech rate for clear speech is roughly 100 words per minute (wpm) and in conversational speech, around 200 wpm (Picheny et al., 1986), other techniques with higher temporal resolution have also been used to investigate the language abilities required to encode speech. One of such techniques is event-related brain potentials (ERPs)–retrieved from the electroencephalogram (EEG)–(Näätänen et al., 2007) which are defined as electrical brain responses time-locked to a specific sensory stimulus (Luck, 2005). Several auditory evoked potentials (AEPs) have been used to explore language impairments (Luck, 2005; Friederici, 2006; Barman et al., 2021). But, recently, an electrophysiological response termed frequency-following response (FFR) have gained interest.

The FFR represents a snapshot into the neural encoding of the temporal and spectral features of the eliciting speech stimulus, allowing to explore the accuracy of neural encoding along the entire auditory system (Coffey et al., 2019; Gorina-Careta et al., 2021). A precise neural tracking of these two frequency components is critical to ensure language acquisition and comprehension (He et al., 2007; Moon and Hong, 2014; Lau et al., 2017). F_0_ is determined by the rate of glottis pulse and its neural encoding provides syntactic, phonological, and semantic cues (Nakatani and Schaffer, 1978), crucial for speaker identification (Mary and Yegnanarayana, 2008) and for dividing the continuous speech into word-form units (François et al., 2017). On the other hand, the temporal fine structure of the speech input, particularly its formants, is relevant for phoneme discrimination (Diehl and Lindblom, 2004; Hornickel et al., 2009) and, indeed, for speech perception in compromised comprehension contexts (Hopkins et al., 2008; Moore, 2008).

The FFR has been recorded in normal and clinical populations at the adulthood (White-Schwoch et al., 2020; Bidelman and Momtaz, 2021), childhood (Thompson et al., 2019, 2021), and the neonatal periods (Gardi et al., 1979; Jeng et al., 2010, 2011, 2016a,b; Ribas-Prats et al., 2019; Richard et al., 2020; Lemos et al., 2021; Ribas-Prats et al., 2022, 2023; see reviews in Gorina-Careta et al., 2022). In premature populations, recent FFR studies disclosed altered neural encoding of several speech cues crucial to ensure proper language acquisition (Madrid et al., 2021; Novitskiy et al., 2023). Madrid et al. (2021) enrolled 28 preterm infants with ≤32 weeks of gestational age (GA) in a longitudinal study. The FFR was recorded at 33, 35, 48–52, and 62–66 weeks of GA, and a decreased onset with age was observed on several FFR components. In the spectral domain, spectral amplitude of the stimulus fundamental frequency (F_0_) and for the lower frequencies of the first formant (F_1_; i.e., <270 Hz) showed an increase with age. However, for higher frequencies of the first formant and higher harmonics (>270 Hz) such increase was not found. Novitskiy et al. (2023) recorded the FFR from 45 preterm infants with a GA ≤ 34 weeks and 45 term infants from 0 to 12 months of age during natural sleep. In this study, three stimuli that differed in pitch were employed although no main effect of stimulus was found. Nevertheless, a main effect of age and prematurity in different FFR parameters related to synchronization and power were reported.

As discussed above, existing FFR studies in premature populations conducted during the first years of life suggest that premature infants born before 34 weeks of GA have limited neural encoding abilities of complex sound features (Madrid et al., 2021; Novitskiy et al., 2023). However, no previous studies explored preterm babies born from 34 0/7 through 36 6/7 weeks of GA. These neonates are defined as late preterm neonates (Raju et al., 2006; Engle et al., 2007) and, although they have higher rates of mortality and morbidity than term neonates (Kramer et al., 2000; Shapiro-Mendoza et al., 2006; Tomashek et al., 2006), understanding of the mechanisms of disease experienced by late-preterm neonates is largely incomplete (Seubert et al., 1999; Escobar et al., 2006; Hunt, 2006; Kinney, 2006; Laptook and Jackson, 2006; Ward, 2006). Hence, our study was set to investigate by means of FFR recordings the neural encoding of two important stimulus characteristics: pitch, quantified as the spectral amplitude to the stimulus fundamental frequency (F_0_), and the stimulus temporal fine structure, which relates to vowel formant composition, measured as the spectral amplitude at the first formant (F_1_) of the stimulus. Also, the FFR’s neural lag was computed. To contribute to explore, differentially, of being born premature from age effects, two terms neonate groups were included: one aged from 37.57 weeks to 39.14 weeks and another aged from 40.14 weeks to 42.29 weeks.

## Materials and methods

### Participants

A total of 36 infants (9 females) were enrolled from the *Sant Joan de Déu Barcelona Children’s Hospital* (Catalonia, Spain) between April 2019 and January 2020. Their gestational age (GA) was from 35.14 to 42.29 weeks. Exclusion criteria were an arterial pH ≤ 7.15 at the time of birth, and an APGAR score < 7 after 5 min of birth, multiple gestations, chromosomal or major structural abnormalities, familiar history of hearing loss and other risk factors associated with hearing impairment (American Academy of Pediatrics, Joint Committee on Infant Hearing, 2019). All infants passed the newborn hearing screening using an automated ABR system (ALGO 3i, Natus Medical Incorporated, San Carlos, CA) as part of standard medical practice. The study was approved by the Bioethics Committee of *Sant Joan de Déu Barcelona Children’s Hospital* (Approval ID: PIC-53-17) and all parents gave their written informed consent in accordance with the Declaration of Helsinki.

Infants were divided into three groups according to their GA with the same ratio of females to males in each group (1,3). Terms neonates were divided according to Lavie et al. (2023): infants born preterm (PRE group, 36.05 ± 0.63 weeks), early term neonates (EARLY group, 38.30 ± 0.57 weeks) and late term neonates (LATE group, 41.01 ± 0.64 weeks). Significant birth weight differences were observed between the three groups (H(2) = 25.377, *p* < 0.0001), and all *Post hoc* pairwise comparisons yielded significant differences [PRE vs. EARLY: mean = 2633.75 g vs. 3021.67 g, *p* = 0.004, 95% CI (69.66, 706.17); PRE vs. LATE: mean = 2633.75 g vs. 3530.00 g, *p* < 0.0001, 95% CI (577.99, 1214.51); EARLY vs. LATE: mean = 3021.67 g vs. 3530.00 g, *p* < 0.001, 95% CI (190.08, 826.59)].

### Stimuli

In the present study, a click stimulus and the consonant-vowel /da/ were presented in alternating polarities at 60 dB SPL to elicit the auditory brainstem response (ABR) and the FFR, respectively. According to Jeng et al. (2011) and see Lemos et al. (2021), both stimuli were delivered to the right ear through ER3C shielded earphones of 300 Ω (Etymotic Research Inc., Elk Grove Village, IL, United States) connected to Flexicoupler® (Natus Medical Incorporated, San Carlos, CA) adaptor.

#### Click stimulus

Auditory brainstem responses (ABRs) to click stimuli were obtained to verify neural integrity in the right ear in all participants (Intelligent Hearing System SmartEP system, IHS, Miami, FL). A square wave click stimulus with a duration of 100 μs was used to elicit an ABR for each neonate analyzed. The stimuli were presented at a rate of 19.30 Hz and a silent inter-stimulus interval of 51.71 ms, based on the procedure followed in previous newborn FFR studies (Ribas-Prats et al., 2019; Arenillas-Alcón et al., 2021, 2023; Ribas-Prats et al., 2022, 2023).

#### /da/ stimulus

To obtain the FFR, the consonant-vowel syllable /da/ was chosen, as this is the most commonly stimulus used in neonatal FFR research (Richard et al., 2020; Lemos et al., 2021; Gorina-Careta et al., 2022). The stimulus fundamental frequency (F_0_) was 113 Hz, chosen to avoid electric power-line frequency interference in Europe at 50 Hz. The stimulus duration was 170 ms, with 10 ms for the onset period, 47 ms for the consonant transition, and 113 ms for the steady vowel section. The presentation rate was 3.7 Hz and the silent inter-stimulus interval was 100.27 ms. In contrast to the stimulus F_0_, which remained at the same frequency along the entire stimulus duration, the first (F_1_) and the second (F_2_) formants of the stimulus varied during the consonant transition, from 553 to 688 Hz and 1,438 to 1,214 Hz, respectively. Both formants at the vowel section remained constant at 688 Hz and 1,214 Hz, respectively.

### Procedure

Stimuli presentation and EEG recordings were conducted with *SmartEP* platform including the *cABR* and *Advanced Hearing Research* modules (Intelligent Hearing Systems, Miami, FL, USA). Disposable Ag/AgCl electrodes were used for each infant and placed according to a vertical montage: active electrode at Fpz location, ground electrode upon the forehead, and references upon mastoids. EEG recording sessions took place in the hospital room while babies slept in their cradle, following the same procedure employed in previous studies with newborns (see Ribas-Prats et al., 2019; Arenillas-Alcón et al., 2021, 2023; Ribas-Prats et al., 2022, 2023).

A sampling rate of 13,333 Hz was used to acquire the continuous EEG. Previous to cut the EEG signal into epochs, recordings were filtered online from 30 to 1,500 Hz. The neural responses to click stimuli were epoched into sweeps of 51.81 ms, including 10.93 ms for the baseline and 40.88 ms for post-stimulus recording period. The epoch for the neural response to /da/ stimulus was 270.27 ms, including 40.95 ms for the baseline and 229.32 ms for post-stimulus recording period. The detection in any sweep of a voltage deflection above ±30 μV resulted in the online rejection as an artifact.

A total of 4000 artifact-free responses to click and to /da/ were analyzed. For the click stimulus, neural responses were acquired in two blocks of 2000 sweeps, and for the /da/ stimulus condition, it was recorded in four blocks of 1,000 sweeps. Data epoching and rejection were performed online. Less than 10% of sweeps were rejected in click or /da/ stimuli blocks in the all sample, respectively. Impedances remained under 10 kΩ during the whole duration of the recording session.

### Data processing

Based on previous studies, only the recordings corresponding to the right ear (ipsilateral to the auditory stimulation) were employed in the statistical analysis (Hornickel et al., 2009; Ribas-Prats et al., 2019; Arenillas-Alcón et al., 2021; Ribas-Prats et al., 2022, 2023). To verify the auditory pathway integrity, the Wave V peak was identified in the ABR recording to click stimulus (Rushaidin et al., 2009) by an automated peak detection algorithm developed in Matlab R2019b (Mathworks). The script output was the highest value of the recorded response within a specified time window according to the age of the sample. Wave V peak latency and amplitudes were retrieved for statistical analysis.

For FFR analysis, the software from Intelligent Hearing Systems (Miami, FL, EEUU) and an off-line bandpass filter from 80 to 1,500 Hz with an infinite slope was applied to the neural responses to /da/ stimulus. The FFR parameters were calculated based on recent FFR tutorials (Krizman and Kraus, 2019) and according to our FFR analysis pipeline (Ribas-Prats et al., 2019; Arenillas-Alcón et al., 2021; Ribas-Prats et al., 2022, 2023). In sum, four FFR parameters were retrieved, three from neural responses to averaged stimulus polarities [(Rarefaction + Condensation)/2] and one from subtracted polarities [(Rarefaction – Condensation)/2].

The first parameter, called Pre-stimulus RMS, was computed to estimate background EEG activity through the pre-stimulus region. The second parameter was the neural lag, defined as the time elapsed between the stimulus onset and the neural phase-locking. Prior to the cross-correlation computation, the /da/ stimulus was resampled and bandpass filtered with a sampling rate of 13,333 Hz and a filter from 80 to 1,500 Hz, according to the neural response parameters. All subsequent parameters were calculated from the onset of the FFR which was defined as the individual neural lag value.

The third and fourth measurements were related to the neural encoding of F_0_ and temporal fine structure (i.e., the harmonics and formants of the stimulus). These parameters were calculated from spectral information in the FFR after the neural response was zero padded and the Fast Fourier Transform (FFT) was applied (Skoe and Kraus, 2010).

To explore the stimulus F_0_ encoding, neural responses to each stimulus polarity were averaged before applying the FFT. On the other hand, to investigate the stimulus temporal fine structure encoding, neural responses to each stimulus polarity were subtracted and the FFT was applied to the subtracted waveforms. Moreover, since the FFR phase-locking bounds (i.e., 1,500 Hz; Krizman and Kraus, 2019) limit the possibility to explore neural encoding of the stimulus F_2_, the temporal fine structure analysis was calculated on the harmonics located at the frequency region of the F_1_. Since F_1_ increases along the consonant transition (from 553 to 688 Hz) while remaining constant during the vowel region (688 Hz), the neural encoding of the stimulus F_1_ was analyzed for the consonant transition (10–57 ms) by averaging the spectral amplitude of the fifth and the sixth harmonics (i.e., HH_5-6_), whereas for the vowel region (57–170 ms, HH_6_) it was equivalent to the spectral amplitude of the sixth harmonic only. Since the spectral content of each stimulus region differs, the neural encoding of the stimulus F_0_ were calculated separately for the consonant transition and for the vowel region.

The spectral amplitude of the neural response for each stimulus region (i.e., the consonant transition and the vowel region) and each frequency peak of interest [i.e., F_0_ = 113 Hz at both the consonant transition and the vowel region; F_1_ = average HH_5_ (565 Hz) and HH_6_ (678 Hz) at the consonant transition region, and F_1_ = HH_6_ (678 Hz) at the vowel region] were calculated. All parameters described were computed with routines provided by Intelligent Hearing Systems (Miami, Fl, EEUU) and scripts developed in our laboratory under Matlab R2019b (Mathworks).

### Statistical analysis

The statistical analysis was performed with IBM SPSS Statistics 23.0 (IBM Corporation, Armonk, NY). Normality was assessed with the Shapiro–Wilk’s test. Parameters normally distributed were analyzed by means of one factor ANOVA with group (PRE; EARLY; LATE) as factor. Those parameters which did not follow a normal distribution were analyzed by Kruskal-Wallis’ H. *Accordingly,* pairwise comparisons were conducted by Bonferroni-corrected t tests or by *Mann*–*Whitney’s U test.* A result was considered significant when *p* < 0.05.

## Results

### Auditory brainstem response

**Figure 1:**
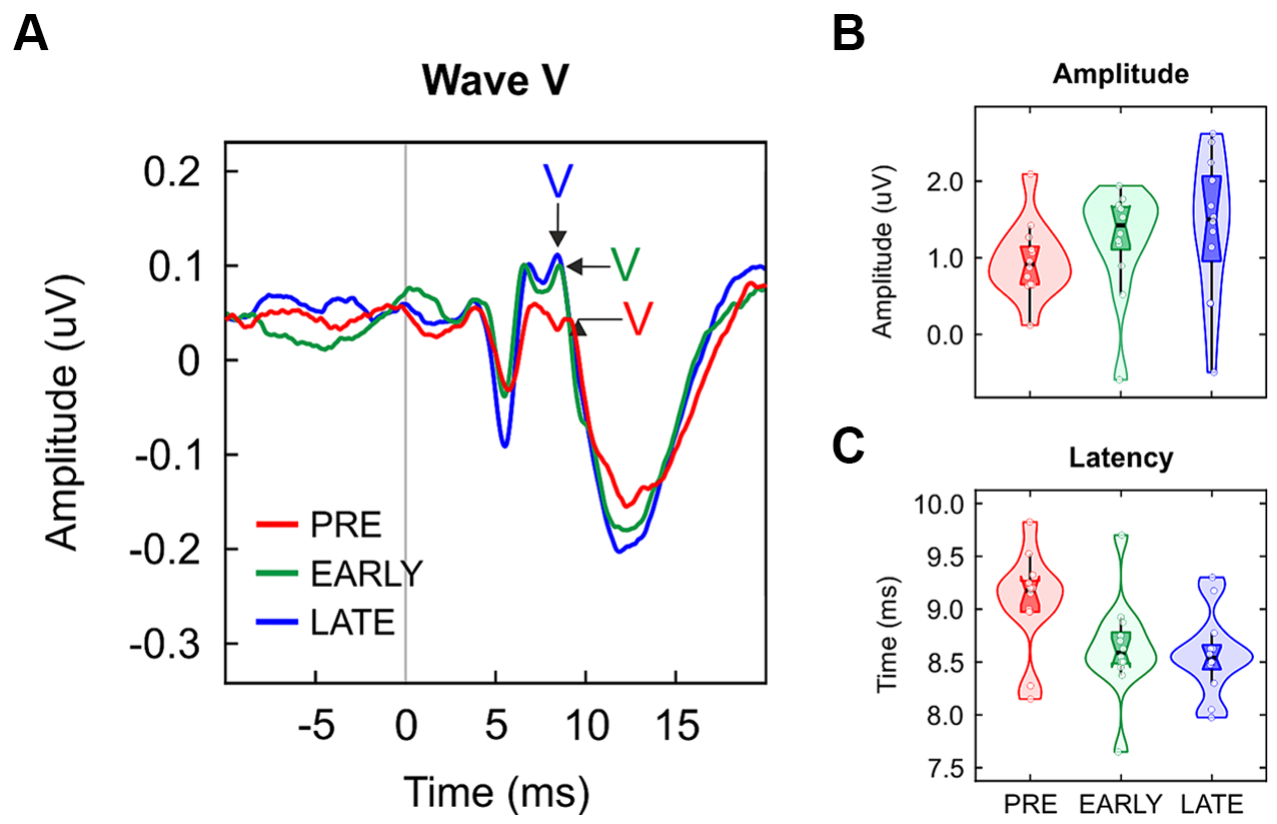
(**A**) Grand-averaged click ABRs for preterm neonates (PRE, red), early at term neonates (EARLY, green) and late at term neonates (LATE, blue), with the wave V peak pointed out. Data distribution (violin plots) for (**B**) wave V amplitude and (**C**) its latency. The black horizontal line illustrates the median and the black vertical line delimits the interquartile range (IQR).

Wave V peak was identified in all preterm, early at term and late at term neonates. Grand-average ABR waveforms are shown in Figure 1A and violin plots of wave V amplitude and latency values are reported in Figures 1B,C, respectively. Wave V amplitude was not significantly different between groups [H(2) = 3.938, *p* = 0.140; Table 1]. Regarding wave V latency, a significant main group effect was found [F(2/33) = 4.506, *p* = 0.019, η^2^ = 0.215; Table 1]. *Post hoc* pairwise comparisons yielded significant differences between PRE and LATE neonates [PRE vs. LATE: mean = 9.07 ms vs. 8.57 ms, *p* = 0.029, 95% CI (0.04, 0.95)].

**Table 1:** Descriptive statistics and comparisons between preterm neonates (PRE), early at term neonates (EARLY) and late at term neonates (LATE) for each wave V and FFR parameter assessed.

Measures	PRE	EARLY	LATE	*t* test	df	*p* value	Effect size
	(*n* = 12)	(*n* = 12)	(*n* = 12)				
Wave V							
Amplitude. μV	0.09 (0.06)^a^	0.14 (0.07)^a^	0.15 (0.16)^a^	3.938*^b^*	2	0.140	
Latency. ms	9.07 (0.47)	8.63 (0.47)	8.57 (0.39)	4.506	2/33	**0.019**	0.215
FFR							
Pre-stimulus, μV	0.02 (0.01)	0.02 (0.01)	0.02 (0.01)	0.074	2/33	0.929	0.004
Neural lag. ms	6.87 (1.15)	6.04 (0.58)	5.63 (0.81)	6.142	2/33	**0.005**	0.271
Spectral amplitude. nV							
Consonant transition (10–57 ms)							
F_0_	26.79 (12.57)	30.04 (10.6)	35.53 (11.63)	1.733	2/33	0.193	0.095
F_1_	0.8 (0.33)	1.07 (0.35)	1.03 (0.37)	2.105	2/33	0.138	0.113
Vowel (57 to 170 ms)							
F_0_	15.92 (4.06)	25.71 (11.16)	24.57 (7.23)	5.342	2/33	**0.010**	0.245
F_1_	0.53 (0.23)	0.62 (0.37)	0.62 (0.27)	0.356	2/33	0.703	0.021

Results are expressed as mean (SD). ^a^Median (IQR, interquartile range). ^**b**^H de Kruskal-Wallis.

### Frequency-following response

**Figure 2:**
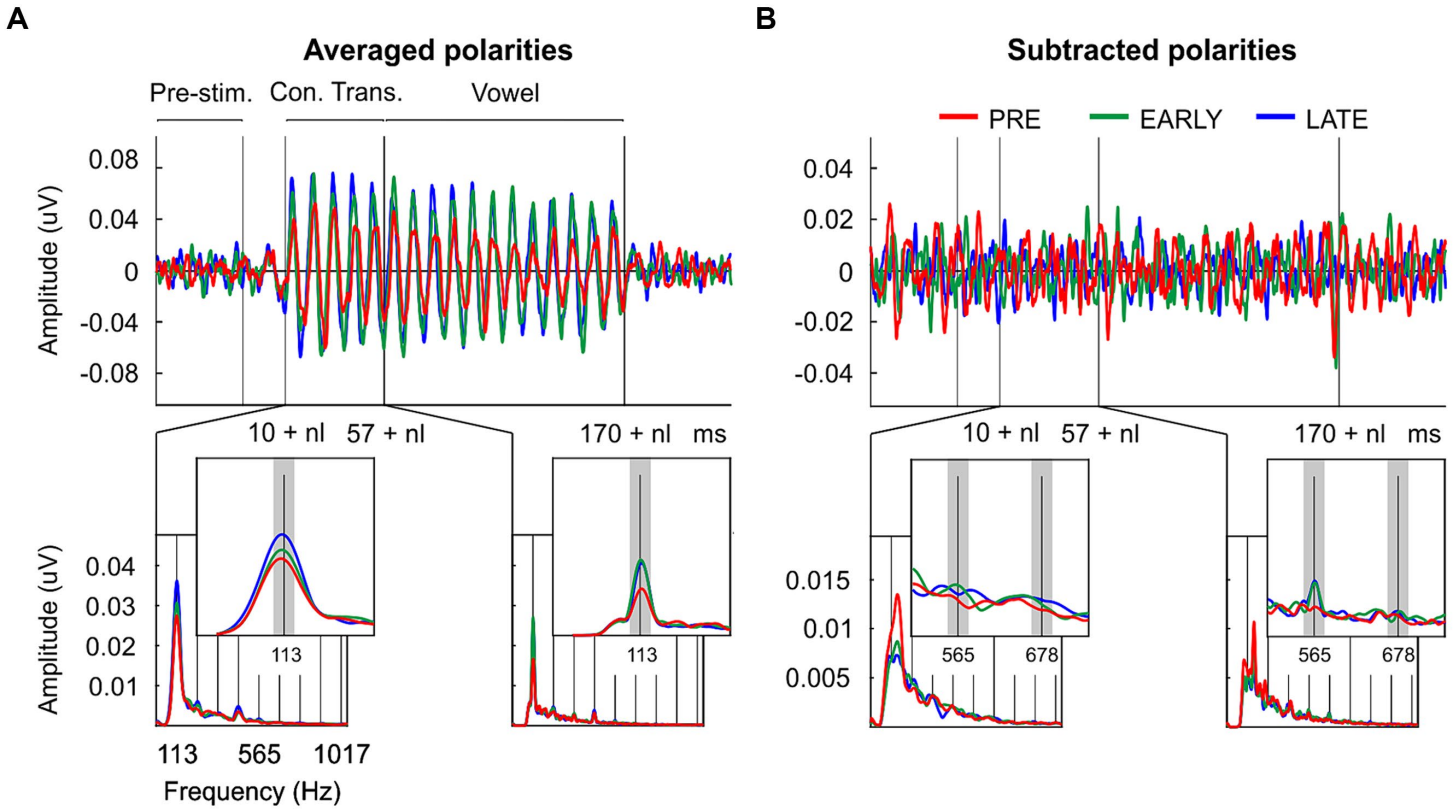
Temporal and spectral neural representation of the consonant-vowel /da/ in the neonate’s auditory brain. **(A)** Grand-averaged FFR waveforms and ampltude FFR spectra extracted from the consonant transition and from the vowel regions after averaged neural response polarities; and **(B)** after subtracted neural response polarities from preterm neonates (PRE, red), early at term neonates (EARLY, green) and late at term neonates (LATE, blue). The signal (s) and noise (n) spectral windows used for FFR quantification are marked with dark and light gray rectangles, respectively.

**Figure 3:**
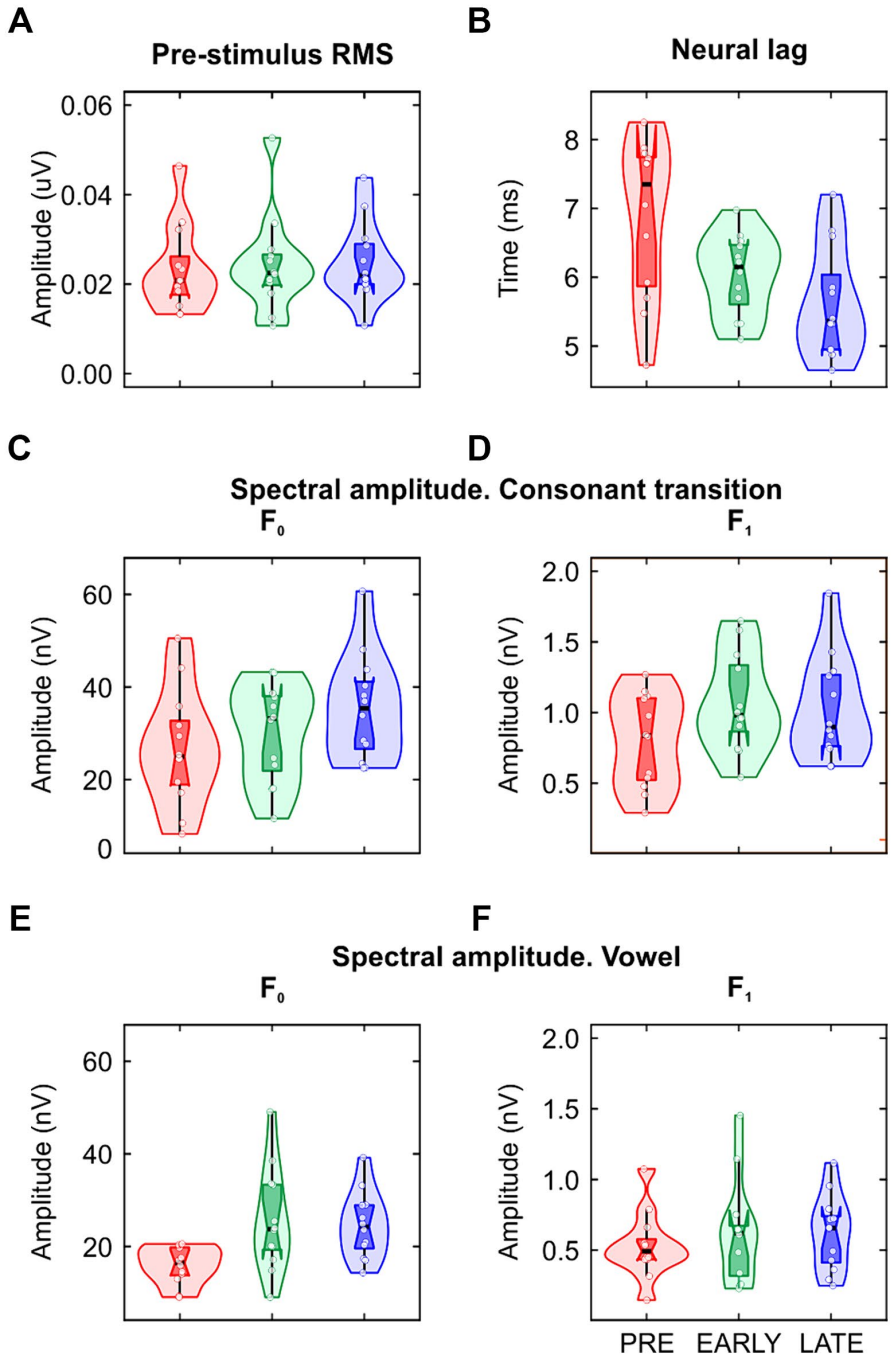
Data distribution (violin plots) of the **(A)** pre-stimulus RMS; **(B)** neural lag; **(C,D)** spectral amplitude at fundamental frequency (F_0_) and first formant (F_1_) extracted from the consonant transition and **(E,F)** from the vowel. The layout is the same to that used in Figures 1B,C.

Clear FFR waveforms were obtained in the three groups (Figure 2). Figure 2A shows the grand-average waveforms for each group for averaged polarities; Figure 2B shows the grand-average waveforms for each group for subtracted polarities. Based on pre-stimulus RMS parameter, similar background EEG activity was observed in the three groups of the study [F(2/33) = 0.074, *p* = 0.929, η_p_^2^ = 0.004; Table 1; Figure 3A]. However, significant differences were found in the response onset as quantified by the neural lag [F(2/33) = 6.142, *p* = 0.005, η_p_^2^ = 0.271; Table 1; Figure 3B]. *Post hoc* pairwise comparisons revealed significant delayed response in the PRE group compared to the LATE group [PRE vs. LATE: mean = 6.87 ms vs. 5.63 ms, *p* = 0.005, 95% CI (0.33, 2.15)].

As described in the data processing section, the FFR was analyzed in each group by calculating the spectral amplitude at the stimulus F_0_ for averaged stimulus polarities (Figure 2A; Figures 3C,E), and at the stimulus F_1_ for subtracted stimulus polarities (Figure 2B; Figures 3D,F; Aiken and Picton, 2008), for each stimulus region of interest: the stimulus consonant transition and the vowel region. Regarding the neural encoding of the stimulus F_0_, no significant differences were found in the spectral amplitudes extracted from the consonant transition [F(2/33) = 1.733, *p* = 0.193, η_p_^2^ = 0.095; Table 1]. However, significant differences emerged at the vowel region [F(2/33) = 5.342, *p* = 0.010, η_p_^2^ = 0.245; Table 1]. A main effect was observed between PRE and EARLY groups [PRE vs. EARLY: mean = 15.92 nV vs. 25.71 nV, *p* = 0.016, 95% CI (−18.05, −1.53)] and between PRE and LATE groups [PRE vs. LATE: mean = 15.92 nV vs. 24.57 nV, *p* = 0.038, 95% CI (−16.92, −0.39)]. The F_1_ neural encoding analysis yielded no significant differences for either the consonant transition [F(2/33) = 2.105, *p* = 0.138, η_p_^2^ = 0.113; Table 1] or the vowel [F(2/33) = 0.356, *p* = 0.703, η_p_^2^ = 0.021; Table 1].

## Discussion

This study reveals functional impairments in the neural encoding of speech sound features in late preterm neonates, by analyzing electrophysiological brain responses elicited to the /da/ syllable. Our results align with previous studies in which an impaired neurodevelopment was associated to being born prematurely (Barre et al., 2011; Nazzi et al., 2015; Carter and Msall, 2017).

Since the earlier a neonate is born, the less likely the neonate is to survive and, in the case of survival to face long-term, health problems and disrupted neurodevelopment (ACOG Practice Bulletin, 2021), most of the clinical studies centered on improving the management of preterm populations are focused on the extreme preterm (<28 0/7 weeks), very preterm (28 0/7–31 6/7 weeks) or moderate (32 0/7–33 6/7 weeks) preterm population. The few clinical reports that compared late preterm infants (34 0/7–36 6/7 weeks) with term infants already suggested the presence of a higher risk for neurodevelopmental handicaps, such as speech disorders (Engle et al., 2007; Karnati et al., 2020). Stene-Larsen et al. (2014) found that late preterm infants were at increased risk for speech and language delays at 18 and 36 months, and Nepomnyaschy et al. (2012) described lower scores in language scales at 2 and 4 years in late preterm infants compared to term infants. In a longitudinal study, Rabie et al. (2015) examined the incidence differences of developmental speech or language disorders in late preterm infants and in term infants, and found an increased proportion of developmental speech and/or language delays in the clinical group.

Given that at 34 weeks of gestation (late-preterm) the fetal brain has reached only about 65% of its development (Kinney, 2006), white matter quintuples its weight from 35 to 41 weeks of gestation (Hüppi et al., 1998), and at this time several cerebral changes crucial for language development take place, such as an exponential neural connectivity growth between the cochlea and the auditory brainstem and its expansion to the auditory cortex (Pujol and Lavigne-Rebillard, 1992; Hepper and Shahidullah, 1994; Hall, 2000), more studies characterizing the abnormal functionality of neural underpinnings of language processing in these cohort of preterm neonates are needed.

Thus, in the present study we used the frequency-following response (FFR) to contribute to this aim. This electrophysiological response has a high temporal resolution allowing to explore one of the crucial processing abilities to ensure the first steps for appropriate language acquisition, namely, the capacity to catch the temporal and spectral characteristics of the speech input (Coffey et al., 2019; Gorina-Careta et al., 2021). Several studies conducted in different age cohorts (Gardi et al., 1979; Jeng et al., 2010, 2011, 2016a,b; Ribas-Prats et al., 2019; Thompson et al., 2019, 2021; White-Schwoch et al., 2020; Bidelman and Momtaz, 2021) and different language impairments (Russo et al., 2008; White-Schwoch et al., 2015, 2020; Otto-Meyer et al., 2018; Font-Alaminos et al., 2021) suggest the potential clinical use of the FFR.

Since language development can be affected by the presence of auditory impairment (Yoshinaga-Itano et al., 1998; American Academy of Pediatrics, Joint Committee on Infant Hearing, 2019), prior to the FFR recording to the /da/ syllable we tested the auditory pathway integrity by identifying the Wave V in ABR, elicited to a click stimulus (Hall, 2006; Sharma et al., 2016). Once the presence of the Wave V was confirmed in every individual participant, we analyzed group differences in the main Wave V parameters. Results revealed a significant latency delay in late preterm neonates compared to term neonates. These results aligned with those from previous studies in which, although different click rate and filter settings were used, a clear delayed Wave V was observed (Despland and Galambos, 1980; Sleifer et al., 2007; for a revision of 14 studies, see Stipdonk et al., 2016). This delay was associated with disruptions in the myelination and synaptogenesis process taking place on the fetal brain of the clinical group with direct affections on the central auditory system (Javel, 1980; Sleifer et al., 2007; Volpe, 2019).

Although ABR and FFR are both electrophysiological responses originating during the first milliseconds after the stimulus onset, their characteristics are considerably different. While the ABR morphology is characterized by a series of peaks related to each station of the auditory pathway from the eighth cranial nerve up to the inferior colliculus (Felix et al., 2018), the FFR morphology depends on the stimulus used to elicit it, becoming a mirror of it. Thus, this electrophysiological response allows us to investigate more precisely how the auditory brain captures the stimulus spectro-temporal components (Krizman and Kraus, 2019).

The FFR analysis of the present study revealed that late preterm neonates, compared to term neonates, exhibit a delayed neural response of complex sounds, as revealed by the neural lag parameter, and an impoverished neural encoding of a specific component of the speech input–its fundamental frequency (F_0_)–, as revealed by means of the spectral amplitude parameter. Regarding the neural lag, Madrid et al. (2021) described a latency decrement with age in preterm neonates. However, the GA of their preterm neonates (i.e., ≤ 32 weeks of GA), as well as the lack of a control group, limits the possibility to compare their results directly with the present findings. Novitskiy et al. (2023) explored whether the neural encoding of three different speech stimuli differed between a group of preterm infants delivered from 22 to 34 weeks of gestation and a group of term infants delivered from 38 to 40 weeks of gestation. To that end, different parameters related to response synchronization and response power were retrieved from the FFR. A main group effect was found for some synchronization parameters, with higher values in the term than in the preterm infants. On the other hand, no group differences were observed in measures related to response power. However, the length of gestation of the preterm sample, the age of the sample and other methodological differences hinders the comparison our results with those of Novitskiy et al. (2023).

The present study contributes to better understanding the variability and significance of FFR responses in different neonatal conditions (Musacchia et al., 2020; Ribas-Prats et al., 2022, 2023). In neonates affected by progressive moderate hyperbilirubinemia, the FFR parallels bilirubin levels, allowing to monitor neurotoxicity decrements resulting from phototherapy (Musacchia et al., 2020). In term small-for-gestational-age, a poor neural encoding of the stimulus F_0_ was observed by means of the normalized spectral amplitude, quantified as SNR at the vowel region, and these neural encoding disruptions were associated with specific white matter affections (Ribas-Prats et al., 2022). In those neonates located at the opposite extreme of the birth weight continuum -a condition known as large for gestational age (LGA)-, smaller spectral amplitudes for the consonant transition and for the vowel were found, and these observations in the LGA group were linked to a disrupted fine-grained central auditory system microstructure due to adipose tissue accumulation. Taking into account the potential clinical implications of the neonatal FFR, we suggest that the implementation of the FFR recordings in the clinical routines as a part of the auditory screening could have a promising impact to minimize cognitive consequences arising from any neonatal medical condition.

We speculate that the present FFR results could be related to disruptions occurring in the fetal brain growth of late preterm neonates, with special affection to synaptogenesis processes and white matter, in particular disruptions in the myelination progression (Javel, 1980; Sleifer et al., 2007; Volpe, 2019). By the 26th week of gestation linear arrays of oligodendrocytes outline the axon of the cochlear nerve and brainstem pathways (Moore et al., 1995). By week 29 this myelination process extended to the entire auditory pathway, including the commissure and the brachium of the inferior culliculus (IC; Moore et al., 1995) which is a primary generator of the FFR (Coffey et al., 2019; Gorina-Careta et al., 2021). From week 29 of gestation onwards, myelination density rises in all pathways and does not reach its maximum until at least 1 year of postnatal age (Moore et al., 1995). The increased density of myelination has been linked to a major synchronized conduction of auditory impulses from the cochlear nerve to the end of the auditory system, and several studies focused on the ABR disclosed a decreased wave V latency associated with these myelination trajectories (Johnson et al., 2008; Sharma et al., 2016). Regarding the FFR, differences in myelinization density between preterm and term neonates could lead to a decreased synchronization in the auditory impulses’ transmission in the clinical group and, thus, to a poorest robustness with which language stimuli are encoded, as observed here. Also, the functionality of the auditory nervous system in the clinical group may also be impaired due to hypoxic episodes occurring with a significant major incidence in late term neonates compared to term neonates during the first days of life (before 43 weeks corrected postconceptional age; Ramanathan et al., 2001; Williams et al., 2019).

On the other hand, the lack of significant results at the consonant transition region could be explained because of the rapid frequency changes occurring during a brief period (i.e., consonant transition length of 47 ms compared to the 113 ms for the vowel region). These fast fluctuations would demand a precise phase-locking at the stimulus F_0_ that is not detected in the neonates’ auditory system, which is still in structural and functional development (Moore and Guan, 2001; Moore and Linthicum, 2007), as it was observed in a previous FFR clinical study in which a group of term neonates affected by fetal growth restriction was compared with a healthy term neonate group (Ribas-Prats et al., 2022).

Regarding the neural encoding of the stimulus F_1_, the lack of group differences supports the existence of a different maturational pattern for each of the speech cues under analysis (i.e., stimulus F_0_ and F_1_; Anderson et al., 2015; Van Dyke et al., 2017; Arenillas-Alcón et al., 2021; Ribas-Prats et al., 2023). In addition, the lack of effects reported in the present study compared to the significant higher improvement in the neural encoding of F_1_ from 1 to 0 months of age recently described by Ribas-Prats et al. (2023), suggests that is the environmental exposure during the perinatal period which may explain the auditory system refinement needed to catch the neural encoding of the stimulus F_1_.

The interest to explore the neural encoding of both the stimulus F_0_ and its F_1_ is because they provide two different angles on speech processing abilities. Indeed, the neural encoding of F_0_ is critical for early language acquisition, as it facilitates the cutting of the continuous speech into the linguistic elements of the discourse (François et al., 2017) and contributes to talker identification, promoting first social interactions (Mary and Yegnanarayana, 2008). On the other hand, the neural encoding of the stimulus F_1_ supports phoneme discrimination (Diehl and Lindblom, 2004; Hornickel et al., 2009) and speech comprehension (Hopkins et al., 2008; Moore, 2008).

Finally, it should be noted that the inclusion of two term neonate groups allowed us to explore age effects, which has been largely described in longitudinal and cross sectional FFR studies (Anderson et al., 2015; Van Dyke et al., 2017; Arenillas-Alcón et al., 2021; Ribas-Prats et al., 2023). Exploring the magnitude of the FFR group differences in each of the parameters analyzed, it is evident that the differences are more abrupt between those neonates born preterm and those neonates born early term, than between neonates born early and those born late term. Thus, this finding suggests that the last weeks of gestation are crucial for brain development and for the white matter formation (Javel, 1980; Sleifer et al., 2007; Volpe, 2019). On the other hand, the lack of differences between early and late term neonates leads to better understanding of how FFR responses stabilize over time in early childhood as it was suggested by Llanos et al. (2017).

While the present study contributes to better understanding the neurodevelopmental limitations associated to late prematurity, further longitudinal studies are crucial, in which the neural encoding of stimulus F_1_ could be explored as the effects of specific social environment variables, since the experience-dependent auditory plasticity by means of the FFR have been widely described (Lau et al., 2017, 2019; Llanos et al., 2017; Reetzke et al., 2018; Ribas-Prats et al., 2023). Also, the presentation of more complex sounds such as the use of background noise (White-Schwoch et al., 2015; Musacchia et al., 2018; Thompson et al., 2019) or even the use of continuous speech in FFR studies could be promising to increase the potential implications for clinical assessments and interventions, the external validity and to give rise to stronger conclusions (Kulasingham et al., 2020; Arenillas-Alcón et al., 2021). As stated in the title of the present research, this study is just a pilot proof of concept on the investigation of FFR in late preterm neonates with the inclusion of two (early and late) term neonates for comparison. Thus, one of the major limitations of the study was the sample size. Another important limitation is the lack of sociodemographic data. To discriminate the effects of prematurity from other relevant variables such as social environment or economic status on different aspects of language encoding, those studies with a larger sample size should include these variables in the statistical analysis as covariates. Hence, future studies with larger samples, including cohorts for each term and preterm group based on the GA classification proposed by the WHO and the American College of Obstetricians and Gynecologists (ACOG Practice Bulletin, 2021; World Health Organization, 2023: for prematurity: extremely preterm, very preterm, moderate, and late preterm; for term deliveries: early term, full term, late term, and post term), are needed to better understand and address the specific needs of each of these preterm populations, thus fostering critical improvements in the current clinical guidelines for preterm infants.

## Conclusion

The present study indicates that late preterm neonates exhibit a delayed neural response to complex sounds and an impoverished neural encoding of the stimulus fundamental frequency compared to term neonates born around the 38 and the 41 weeks of gestational age. Our results disclosed specific effects in the vowel region only and are compatible with the myelination and synaptogenesis affections observed in late preterm neonates. Yet, further studies are to be conducted to test whether the neural encoding affections disclosed here by means of the FFR could be causing a ‘knock-on effect’: a dysfunction at birth that may point to the beginning of a developmental problem that increases with age.

## Data availability statement

The raw data supporting the conclusions of this article will be made available by the authors, without undue reservation.

## Ethics statement

The studies involving humans were approved by Bioethics Committee of Sant Joan de Déu Barcelona Children’s Hospital. The studies were conducted in accordance with the local legislation and institutional requirements. Written informed consent for participation in this study was provided by the participants’ legal guardians/next of kin.

## Author contributions

TR-P: Conceptualization, Data curation, Formal analysis, Investigation, Methodology, Software, Visualization, Writing – original draft, Writing – review & editing. SA-A: Data curation, Investigation, Supervision, Visualization, Writing – review & editing. SM: Conceptualization, Supervision, Writing – review & editing. MG-R: Conceptualization, Supervision, Validation, Writing – review & editing. CE: Conceptualization, Funding acquisition, Investigation, Project administration, Resources, Supervision, Validation, Writing – review & editing.
